# Identification and Validation of Prognostic Related Hallmark ATP-Binding Cassette Transporters Associated With Immune Cell Infiltration Patterns in Thyroid Carcinoma

**DOI:** 10.3389/fonc.2022.781686

**Published:** 2022-06-28

**Authors:** Lidong Wang, Xiaodan Sun, Jingni He, Zhen Liu

**Affiliations:** ^1^ Department of General Surgery, Shengjing Hospital of China Medical University, Shenyang, China; ^2^ Postdoctoral Research Workstation, Jilin Cancer Hospital, Changchun, China; ^3^ Department of 1st Gynecologic Oncology Surgery, Jilin Cancer Hospital, Changchun, China

**Keywords:** thyroid carcinoma, ATP-binding cassette transporters, prognosis, immune cell infiltration, immunomodulation

## Abstract

ATP-binding cassette (ABC) transporters are a large superfamily of membrane proteins that facilitate the translocation of heterogeneous substrates. Studies indicate that ABC transporters may play important roles in various carcinomas. However, the correlation between ABC transporters and immunomodulation in thyroid carcinoma (TC), as well as the prognoses for this disease, is poorly understood.TC data from The Cancer Genome Atlas (TCGA) database were used to identify prognostic hallmark ABC transporters associated with immune cell infiltration patterns *via* multiple bioinformatic analyses. Thereafter, quantitative real-time polymerase chain reaction (qRT-PCR) was performed to validate the expression of these selected hallmark ABC transporters in both TC and para-cancerous thyroid tissues. Of a total of 49 ABC transporters, five (*ABCA8*, *ABCA12*, *ABCB6*, *ABCB8*, and *ABCC10*) were identified as hallmark ABC transporters. All five were differentially expressed in TC and associated with the relapse-free survival rates of patients with TC. Immunoregulation by these five hallmark ABC transporters involved the modulation of various aspects of immune cell infiltration, such as hot or cold tumor subsets and the abundances of infiltrating immune cells, as well as specific immunomodulators and chemokines. Besides the diverse significantly correlated factors, the five hallmark ABC transporters and correlated genes were most highly enriched in plasma membrane, transporter activity, and transmembrane transport of small molecules. In addition, many chemicals, namely bisphenol A and vincristine, affected the expression of these five transporters. The qRT-PCR results of collected TC and para-cancerous thyroid tissues were consistent with those of TCGA. The findings in this study may reveal the role played by these five hallmark ABC transporters in regulating immune cell infiltration patterns in TC as well as the molecular mechanisms underlying their functions, leading to a better understanding of their potential prognostic and immunotherapeutic values.

## Introduction

Thyroid carcinoma (TC) is the most common endocrine malignancy worldwide, and its global incidence rate has been growing over the last three decades. Before the 1990s, the incidence of TC in the United States was relatively stable at approximately 5/100,000. However, its incidence had tripled (15/100,000) by 2014 (1). The incidence of TC in Canada from 2012–2016 was reportedly 17.4/100,000, a figure closely similar to that of the United States (2). Such dramatic increases in the incidence of TC have also been observed in many other countries, including 29 European countries and China (3, 4). According to Global Cancer Statistics, 586,202 new TC cases were reported worldwide in 2020, amounting to an incidence of 13.2/100,000 (5). However, the mortality rate of TC has remained relatively low and stable (<1/100,000); (1–4, 6). The etiology of TC remains unclear. Exposure to ionizing radiation during childhood is considered to be risk factor that is most and closely associated with TC (7). Moreover, other factors, such as chromosomal (genetic) alterations and obesity, are reportedly associated with the occurrence and development of TC (8, 9).

Based on histological features, TC is mainly divided into the following four types: papillary thyroid carcinoma (PTC); follicular thyroid carcinoma (FTC); anaplastic thyroid carcinoma (ATC); and medullary thyroid carcinoma (MTC); (10). PTC represents the most common differentiated subtype of TC, and its incidence has reportedly increased over the past decade (11). Most PTC patients receive favorable prognoses involving 10-year survival rates ranging from 93–97% (12, 13). The 10-year survival rates for FTC and MTC are 85% and 75%, respectively, and thus worse than that for PTC (12). ATC begets the worst prognosis, with a 10-year survival rate of 14% and a median survival of six months (12, 14). Although the majority of TCs, which remain indolent, are associated with an innocuous clinical course, some cases manifest aggressive behavioral patterns, such as metastasis and recurrence, resulting in poorer prognoses. The recurrence rate of PTC following conventional treatment is reportedly as high as 28% (15). Metastasis in TC, which mostly involves the cervical lymph system, acts as an unfavorable factor which leads to poor prognoses (16). The 8th edition of the American Joint Committee on Cancer (AJCC) tumor-node-metastasis (TNM) staging system identifies sex, age, N classification, pathological subtype, and radioactive iodine avidity as some of the major factors affecting the prognosis of PTC (17). In addition, the 10-year cancer-specific survival rate for IVb stage PTC patients over 55 years old presenting with extensive extrathyroidal extension is reportedly 33.3% (18). The limited number of alternative therapeutic strategies that have been used against surgically inoperable and radioiodine-refractory TC have not been successful at improving the survival of TC patients. Therefore, development of new intervention strategies against TC are felt to be warranted.

The tumor microenvironment (TME), which is composed of the extracellular matrix, stromal cells, immune cells, and some secreted factors, plays an important role in TC (19). Of these, tumor immune cell infiltration has been demonstrated to be closely associated with TC progression and prognosis (20, 21). Tumors may be categorized as immunologically hot or cold types, which definitions are based on the degree of immune infiltration in the TME (22). The hot type is characterized by high tumor immunity, indicating association with a stronger immune response and a better survival outcome. By contrast, the TME of a cold tumor is much more immunosuppressive, responding poorly to treatment (23). Various immune cells that infiltrate tumors may either accelerate or decelerate tumor progression, depending on population and activation status (24). Tumor-promoting immune cells, including dendritic cells (DCs), macrophages, and monocytes, may promote tumor growth, metastasis, and drug resistance in the TME, whereas antitumor immune cells, namely B cells, natural killer (NK) cells, and CD8^+^ T cells, suppress tumor cell proliferation, invasion, adhesion, and metastasis. DCs, which play a key role in antigen presentation and cytokine secretion, are increased in TC (25). In TC, infiltrating DCs activate T cells and NK cells *via* tumor antigen presentation. Moreover, these infiltrating DCs are known to produce some immunosuppressive cytokines that inhibit immune responses (26). Tumor-associated macrophages (TAMs), which originate from monocytic precursors, infiltrate into the tumor stroma, and facilitate macrophage polarization from the antitumor M1 phenotype to the tumor-promoting M2 phenotype, thereby aggravating TC growth and metastasis (27–29). Monocytes that differentiate in the bone marrow are mainly responsible for inflammation. Increased monocyte infiltration in a mouse TC model promotes tumor progression by elevating immune-related gene and cytokine expression (30). These results show that immunosuppressive cells in the TME of TC can strengthen the ability of tumor cells to fight immune response, thereby enhancing immune escape. Therefore, investigating immune cell infiltration in relation to regulatory mechanisms of TC may be vital for developing new immunotherapeutic strategies that improve TC patient outcomes.

ATP-binding cassette (ABC) transporters are a large superfamily of membrane proteins that acquire energy from ATP hydrolysis to facilitate the translocation of heterogeneous substrates (31). A total of 49 human ABC transporters are grouped into seven distinct subfamilies as follows: ABCA; ABCB; ABCC; ABCD; ABCE; ABCF; and ABCG (32). All ABC transporters are composed of transmembrane domains (TMDs) and nucleotide-binding domains (NBDs); (33). ATP hydrolysis which occurs at the NBDs, induces conformational changes in TMDs, which, in turn, facilitate inward or outward transportation of specific substrates across the membrane (34). ABC transporters, which are ubiquitous, have been found to be associated with diverse biochemical and physiological processes, such as maintenance of cellular environments, protection from harmful materials, and modulation of drug kinetics (35–37). ABC transporters reportedly play vital roles in numerous carcinomas (38). For example, *ABCG1*, which is overexpressed in clear cell renal cell carcinoma, has been found to be associated with overall patient survival, indicating its potential as a diagnostic and prognostic biomarker in clear cell renal cell carcinoma (39). *ABCB1* and *ABCG2* reportedly play a critical role in the prevention of chemo-resistant liver cancer stem cell death in hepatocellular carcinoma (40). Differentially expressed *ABCC2* and *ABCC5* are considered as diagnostic biomarkers of lung adenocarcinoma, while *ABCC2*, *ABCC6*, and *ABCC8* are reportedly associated with its prognosis (40). Many ABC transporters which are differentially expressed between colorectal cancer (CRC) and non-neoplastic control tissues, may be linked to both the onset and treatment outcomes of CRC (41). The functions of ABC transporters known to be involved in immunity against infection and cancer have been summarized and reviewed, providing a broader understanding of the effects of ABC transporters on immunity to viruses and tumors (42). However, the prognostic roles and immune-related mechanisms of ABC transporters in TC remain unclear.

In this study, we aim to predict the prognostic implications and immune cell infiltration related features of ABC transporters by performing comprehensive analyses followed by validation *via* RT-PCR. Here, we attempt to provide a deeper insight into the immune cell infiltration patterns seen in TC as well as to identify some potential prognostic and immunotherapeutic targets in TC.

## Materials and Methods

### Pre-Processing of Public Data Sources

High-throughput RNA sequencing (RNA-seq) data of 502 TC and 58 normal thyroid tissues obtained from The Cancer Genome Atlas (TCGA) database were considered as the public data source for the purposes of this study. The RNA-seq data in a fragments per kilobase per million format were converted into a transcript per million (TPM) reads format and log2 transformed. The clinical features of TC patients obtained from TCGA dataset are summarized (**Table 1**).

**Table 1:** Clinical features of TC patients included in the study.

Characteristic	Number (%)	
	TCGA	Collected
Total	502 (100)	45 (100)
Gender		
Female	367 (73.1)	35 (77.8)
Male	135 (26.9)	10 (22.2)
Age		
< 55	335 (66.7)	32 (71.1)
≥ 55	167 (33.3)	13 (28.9)
Histological type		
Classical	356 (70.9)	45 (100)
Follicular	101 (20.1)	0 (0.0)
Other	9 (1.8)	0 (0.0)
Tall cell	36 (7.2)	0 (0.0)
T stage		
T1	143 (28.5)	34 (75.5)
T2	164 (32.7)	8 (17.8)
T3	170 (33.9)	3 (6.7)
T4	23 (4.6)	0 (0.0)
Tx	2 (0.3)	0 (0.0)
N stage		
N0	229 (45.6)	27 (60.0)
N1	223 (44.4)	18 (40.0)
Nx	50 (10.0)	0 (0.0)
M stage		
M0	282 (56.2)	45 (100)
M1	9 (1.8)	0 (0.0)
Mx	211 (42.0)	0 (0.0)
Pathologic stage		
I	281 (56.0)	38 (84.4)
II	52 (10.4)	7 (15.6)
III	112 (22.3)	0 (0.0)
IV	55 (10.9)	0 (0.0)
NR	2 (0.4)	0 (0.0)

NR, Not reported.

### Patients and Specimens

A total of 45 TC patients who received surgical therapy at the Shengjing Hospital of China Medical University were selected for the study. The exclusion and inclusion criteria were similar to those of a previously reported study of ours (43). Both TC and para-cancerous thyroid tissues were collected. A para-cancerous thyroid tissue is defined as a tissue situated at least 2 cm far away from the TC area, as confirmed without TC cells by pathologists. Based on postoperative pathological diagnoses, all included TC tissue specimens were the PTC histological type. Informed consent was obtained from all patients. This study was approved by the Ethics Committee of Shengjing Hospital of China Medical University. The clinical features of all collected patients were summarized (**Table 1**). All specimens were immediately stored until needed for total RNA extraction, qRT-PCR and hematoxylin-eosin staining.

### Identification of Hallmark ABC Transporters in TC

We compared the expression levels of all ABC transporters known to be active in TC as well as in normal thyroid tissues, that were available in the public TCGA database. R software, version 3.6.3, with the ggplot2 package (version 3.3.3), was used for this comparison. Correlation between the expression levels of individual genes and prognoses was analyzed *via* the online database Kaplan–Meier plotter (44). To analyze the relapse-free survival (RFS), TC patient samples from TCGA were split into two groups by the relative expression levels of individual genes and assessed by a Kaplan-Meier survival plot. The best performing threshold was considered as the best cutoff value. The hazard ratio (HR) with 95% confidence intervals (CIs) and log rank *P*-value of individual genes were performed. Differentially expressed, prognostic ABC transporters were selected as hallmark transporters for further analysis.

### Immune-Associated Analysis

Immune and stromal scores *via* ESTIMATE (Estimation of STromal and Immune cells in MAlignant Tumor tissues using Expression data) were used to calculate the levels of infiltrating immune and stromal cells (45). The ESTIMATE score is equal to the sum of immune and stromal scores. The abundance of 24 immune cell type in different kinds of tumors can be estimated using gene expression levels obtained from datasets, such as RNA-seq and microarray data, which are calculated *via* the Immune Cell Abundance Identifier (ImmuCellAI); (46). Therefore, the abundance of tumor-infiltrating immune cells in TC and normal thyroid tissues was determined and compared using ImmuCellAI. The correlation between the expression levels of hallmark ABC transporters and the abundance of gene markers of immune cells infiltrating TC tissues were adjusted for corresponding tumor purity and assessed using Tumor IMmune Estimation Resource 2.0 (TIMER 2.0); (47–49). In addition, immunomodulators and chemokines were compared against the expression of each hallmark ABC transporter using an integrated repository portal for tumor–immune system interactions (TISIDB), in order to analyze the correlation between them (50).

### Significant Correlation Analysis and Interaction Network Construction

LinkedOmics is a publicly available platform that includes multi-omics data of 32 TCGA cancer types, and supports multi-omics analysis in a cancer type or pan-cancer analysis. (51). In the LinkFinder modules of LinkedOmics, genes and microRNAs (miRNAs), that were significantly associated with each hallmark ABC transporter, were analyzed statistically using Pearson’s correlation coefficient and presented in both volcano plots and heat maps. In the LinkInterpreter modules of LinkedOmics, transcription factor (TF) targets, that were significantly associated with each hallmark ABC transporter, were enriched through Gene Set Enrichment Analysis. The rank criterion was P-value < 0.05, the minimum number of genes (Size) was 3, and the simulations was 500. The GeneMANIA prediction algorithm is an interface and a large database that can be utilized to analyze gene functions and build an interaction network (52). We predicted the functions of hallmark ABC transporters and 100 resultant closely associated genes. Thereafter, a regulation network was constructed for visualization.

### Enrichment Analysis

We chose FunRich software (version 3.1.3) to perform enrichment analysis of these five hallmark ABC transporters with the top 100 genes that were closely related to them. Prediction of the functional enrichment of these genes was based on four aspects: cellular component; molecular function; biological process; and biological pathway (53). Gene set analysis of these five hallmark ABC transporters involved in cancer-related pathway activities was performed in GSCALite (54). The following cancer-related pathways were included: TSC/mTOR; RTK; RAS/MAPK; PI3K/AKT; hormone ER; hormone AR; epithelial–mesenchymal transition (EMT); DNA damage response; cell cycle; and apoptosis pathways.

### Chemical–Gene–Disease Correlation Analysis

The Comparative Toxicogenomics Database (CTD, version 16548) provides manually curated information regarding chemical–gene, chemical–disease, and gene–disease relationships. This information helps understand the effects exerted by environmental factors on human health (55). The interaction between these five hallmark ABC transporters and chemicals in TC was inferred *via* curated chemical–gene and chemical–disease associations.

### Prognostic Value Analysis

The prognostic value of these five hallmark ABC transporters in TC patients was determined *via* receiver operating characteristic (ROC) curve analysis. ROC analysis was performed using R software (version 3.6.3) with the pROC (version 1.17.0.1) and ggplot2 (version 3.3.3) packages. The area under the curve (AUC) ranges between 0.5 and 1, with an AUC value closer to 1 indicating a better prognostic effect, particularly a longer RFS (AUCs of 0.5–0.7; 0.7–0.9; and AUC > 0.9 indicate low accuracy; moderate accuracy; and high accuracy, respectively).

### Total RNA Extraction and qRT-PCR

Total RNA was extracted from tissues using Trizol (Takara, Dalian, China), and cDNA was synthesized *via* reverse transcription using a PrimeScript™ RT reagent Kit with gDNA Eraser (Takara, Dalian, China). Thereafter, qRT-PCR analysis was performed on a Roche LightCycler 480 II system using a TB Green^®^Premix Ex Taq^™^II kit (Takara, Dalian, China), according to the manufacturers’ protocols. Primer sequences are listed (**Table 2**). The housekeeping gene *glyceraldehyde-3-phosphate dehydrogenase* (*GAPDH*) was used as an internal control. The relative expression of target genes was determined using the 2^-ΔΔ^ CT method, which was similar to that of a previously reported study of ours (43).

**Table 2:** Primers used in this study.

Gene	Primer sequence	Product size (bp)
*ABCA8*	Forward:5’-TCCTTGCTCCTGGACAACAACC-3’ Reverse: 5’-GCTATGTTCTGGTGCTCCACAG-3’	112
*ABCA12*	Forward:5’-CGGCATTTCAGATACCACCGTG-3’ Reverse: 5’-CAGGAGTTGAGATGCCATTGGC-3’	137
*ABCB6*	Forward:5’-GTTCTTCAACGCCTGGTTTGGC-3’ Reverse: 5’-AGCACGACGAAACTTGGTTCTCC-3’	103
*ABCB8*	Forward:5’-CCTGCTTATCCTCTATGGTGTCC-3’ Reverse: 5’-GCCCTGTCTTATTGGCGTCAAAG-3’	158
*ABCC10*	Forward:5’-TCCAGTTTGCCACCATCCGAGA-3’ Reverse: 5’-ACCTCTGTCTGGTCTCCAGCAG-3’	133
*GAPDH*	Forward: 5’-GCACCGTCAAGGCTGAGAAC-3’ Reverse: 5’-TGGTGAAGACGCCAGTGGA-3’	138

### Hematoxylin-Eosin Staining

Paraffin-fixed sample sections with 3 μm thick were prepared. Briefly, slides were dewaxed and rehydrated, then nuclei were stained with hematoxylin and cytoplasm was stained with eosin. After dehydrating, slides were mounted with neutral balsam. The images were photographed by a microscope.

### Statistical Analysis

Student’s *t*-tests or Wilcoxon rank-sum tests were used to compare between the levels of gene expression in TC and normal tissues. Log-rank test and the Kaplan–Meier method were used to depict survival curves. Pearson’s correlation coefficient was selected to analyze significantly correlated genes. Spearman’s rank correlation coefficient was used to analyze the results of immune-associated analyses. Statistical significance was set at *P* < 0.05.

## Results

### Selected Hallmark ABC Transporters Were Differentially Expressed and Correlated With TC Progression

**Figure 1:**
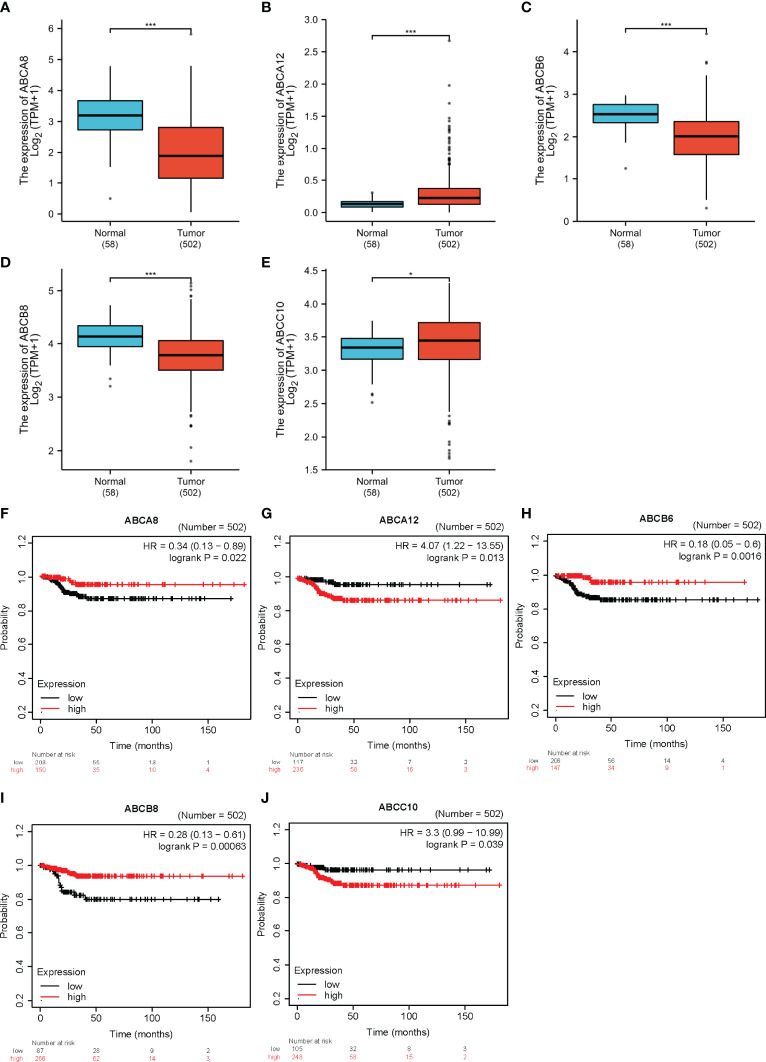
The expression levels and prognostic values of five hallmark ABC transporters in TC. **(A–E)** *ABCA8*, *ABCB6*, and *ABCB8* mRNA expression was downregulated in TC compared with that in normal tissues, while that of *ABCA12* and *ABCC10* was upregulated. **(F–J)** Correlation between expression of the five hallmark ABC transporters and prognoses for TC. *P < 0.05, ***P < 0.001.

To determine the expression and significance of ABC transporters in TC, we compared the transcription levels of all ABC transporters in TC and normal thyroid tissues obtained from the TCGA database. We also analyzed the correlation between the expression levels of all ABC transporters and TC prognoses using the Kaplan–Meier plotter. An integrated comparison indicated that five ABC transporters, namely *ABCA8*, *ABCA12*, *ABCB6*, *ABCB8*, and *ABCC10*, were differentially expressed. In addition, these five were also significantly associated with the RFS of TC. As such, these five were considered as hallmark ABC transporters (*P* < 0.05; **Figure 1**). Among these hallmark genes, *ABCA12* and *ABCC10* showed significantly higher expression in TC and were associated with a worse RFS as well, indicating that these played a prominent role in promoting TC progression. Moreover, *ABCA8*, *ABCB6*, and *ABCB8* showed significantly lower expression levels in TC, in addition to being associated with a worse RFS, demonstrating that these three mainly functioned as inhibitors of TC progression. Therefore, we subsequently analyzed these five hallmark ABC transporters in order to elucidate the molecular mechanisms underlying the role played by them in TC progression.

### Immune-Associated Analysis of Hallmark ABC Transporters in TC

**Figure 2:**
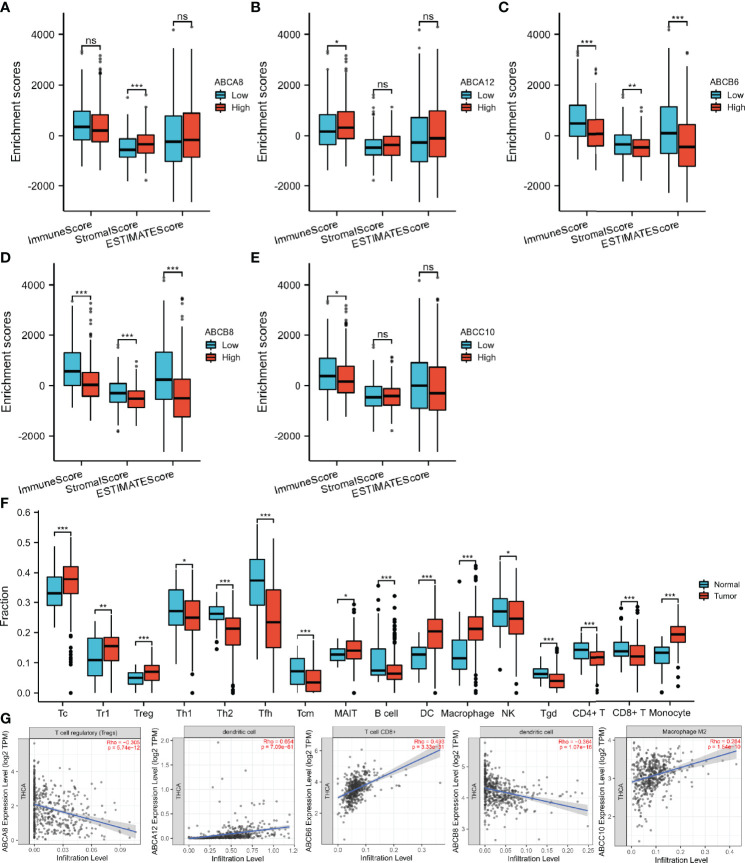
Immune-associated analysis of the hallmark ABC transporters in TC. **(A–E)** Correlation between the expression levels of hallmark ABC transporters and immune, stromal, and ESTIMATE scores. **(F)** Differential abundances of tumor-infiltrating immune cells between TC and normal thyroid tissues. **(G)** Correlation between the expression levels of hallmark ABC transporters and the abundances of tumor-infiltrating immune cells, which were most associated with each hallmark ABC transporters. *P < 0.05, **P <0.01, ***P < 0.001. ns, not significant..

Since immunomodulation plays a vital role in TC progression, we investigated the correlation between immune cell infiltration and the expression levels of the five selected hallmark ABC transporters in TC. Firstly, we evaluated enrichment differences between immune, stromal, and ESTIMATE scores based on the expression of the five hallmark ABC transporters, respectively. The results revealed that the expression levels of all five hallmark ABC transporters were associated with at least one of the immune, stromal, and ESTIMATE scores of TC obtained from TCGA database (**Figures 2A-E**). Especially, both *ABCB6* and *ABCB8* were related to all three types of scores. Thereafter, we investigated the differences between the infiltration of 24 immune cell types in TC and normal thyroid tissues using ImmuCellAI. Among them, a total of 16 types of immune cells were discovered to be differentially infiltrated between TC and normal thyroid tissues, indicating that they may be performing immunoregulatory functions in the progression of TC. These results showed that the abundances of cytotoxic T cells (Tc), type 1 T regulatory cells (Tr1), regulatory T cells (Treg), mucosal-associated invariant T cells, DCs, macrophages, and monocytes in TC tissues were higher than those in normal thyroid tissues. Meanwhile, the abundances of T helper 1 cells (Th1), Th2, follicular helper T cells (Tfh), central memory T cells (Tcm), B cells, NK cells, gamma delta (γδ) T cells (Tgd), CD4^+^ T cells, and CD8^+^ T cells in TC tissues were decreased (**Figure 2F**). Thereafter, we used TIMER 2.0 to explore the correlation between the expression levels of hallmark ABC transporters and the abundances of gene markers of differentially infiltrated immune cells in TC. All five hallmark ABC transporters were associated with Th2 cells, Tcm cells, Treg cells, monocytes, and DCs. Of all the expression levels of these transporters, the expression level that was most significantly negatively correlated with the abundance of Tregs (rho = −0.305, *P* < 0.001) was that of *ABCA8*. In addition, the results showed that *ABCA8* expression was associated with most gene markers of infiltrated immune cells, except those of Th1 cells and monocytes. In addition to being the expression level that was most positively correlated with DCs (rho = 0.654, *P* < 0.001), the expression of *ABCA12* was correlated with nearly all infiltrated immune cells, except M1 macrophages. *ABCB6* expression, which showed the most positive correlation with CD8^+^ T cells (rho = 0.493, *P* < 0.001), appeared to be negatively correlated with most gene markers, except with NK. *ABCB8* expression, which showed the most negative correlation with DCs (rho = −0.364, *P* < 0.001), showed no correlation with Th2 cells, Tfh cells, or NK cells. *ABCC10* expression showed the highest positive correlation with the abundance of M2 macrophages and most gene markers, except with that of NK cells and DCs (rho = 0.284, *P* < 0.001); (**Table 3**; **Figure 2G**).

**Table 3:** Transcription factor targets of the five hallmark ABC transporters in TC.

ABC family	Transcription factor target	Enrichment ratio	FDR
*ABCA8*	V$LXR_DR4_Q3	1.3328	0.0042905
	V$MZF1_01	1.2438	0.0016919
	V$FOX_Q2	1.2433	0.0016919
	V$AHRARNT_01	1.2431	0.011955
	V$CMYB_01	1.2150	0.0028618
*ABCA12*	V$NFKB_C	1.1400	0.044392
	RGAGGAARY_V$PU1_Q6	1.1116	0.018903
	RYTTCCTG_V$ETS2_B	1.0815	0.010427
	TGANTCA_V$AP1_C	1.0725	0.018903
*ABCB6*	V$ELK1_02	1.1574	0.019029
	V$E2F_Q4_01	1.1382	0.047158
	GTGACGY_V$E4F1_Q6	1.0871	0.045609
	SCGGAAGY_V$ELK1_02	1.0822	0.0021504
*ABCB8*	V$ELK1_02	1.1567	0.0035876
	V$YY1_02	1.1456	0.0073703
	V$NRF1_Q6	1.1425	0.0092683
	V$NRF2_01	1.1373	0.0083046
	SCGGAAGY_V$ELK1_02	1.1214	6.2184e-10
*ABCC10*	V$PAX8_01	1.4316	0.011985
	V$SP1_Q6	1.2090	0.0016837
	V$MYCMAX_B	1.1981	0.0022301
	V$NFKAPPAB65_01	1.1864	0.0061827
	V$NFKB_C	1.1806	0.0051293

FDR, false discovery rate.

**Figure 3:**
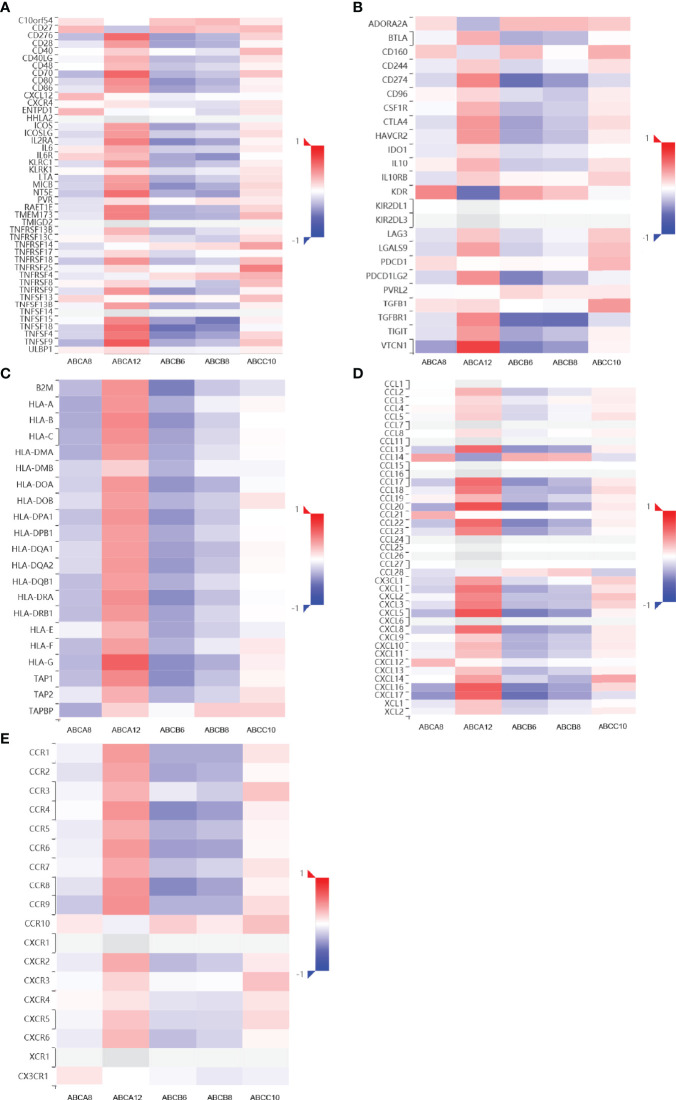
Immunomodulators and chemokines-associated analysis of the hallmark ABC transporters in TC. **(A–C)** Correlation between the expression levels of hallmark ABC transporters and immunomodulators in TC. **(D, E)** Correlation between the expression levels of hallmark ABC transporters and chemokine ligands and receptors in TC.

Moreover, we analyzed the relationship between immunomodulators and the expression levels of hallmark ABC transporters using TISIDB (**Figures 3A-C**). Of all five expression levels, that of *ABCA8* showed the most negative correlation with *VTCN1* (rho = −0.369, *P* < 0.001) as well as the most positive correlation with *KDR* (rho = 0.467, *P* < 0.001). *ABCA12* expression had the most positive correlation with *VTCN1* (rho = 0.755, *P* < 0.001) and the most negative correlation with *KDR* (rho = −0.540, *P* < 0.001). *ABCB6* expression showed a weak to moderate negative correlation with most immunomodulators, particularly *CD274* (rho = −0.550, *P* < 0.001), *TGFBR1* (rho = −0.546, *P* < 0.001), and TNFSF18 (rho = −0.545, *P* < 0.001). Similarly, the expression levels of *ABCB6*, and *ABCB8* showed a weak to moderate negative correlation with most immunomodulators, particularly *TGFBR1* (rho = −0.559, *P* < 0.001) and *TNFSF15* (rho = −0.518, *P* < 0.001). Moreover, the highest positive correlation with *TNFRSF25* (rho = 0.499, *P* < 0.001) was shown by *ABCC10* expression. Finally, we analyzed the association between the five hallmark ABC transporters and 41 types of chemokine ligands and 18 types of receptors (**Figures 3D, E**). The results showed that *ABCA8* expression had the most positive correlation with *CCL14* (rho = 0.359, *P* < 0.001) and the most negative correlation with *CXCL17* (rho = −0.406, *P* < 0.001). *ABCA12* expression showed a moderate to strong positive correlation with most ligands and receptors, particularly *CCL20* (rho = 0.684, *P* < 0.001), *CXCL5* (rho = 0.670, *P* < 0.001), *CXCL16* (rho = 0.638, *P* < 0.001), and *CCR9* (rho = 0.431, *P* < 0.001). *ABCB6* expression showed a weak to moderate negative correlation with most ligands and receptors, including *CXCL17* (rho = −0.546, *P* < 0.001), *CXCL5* (rho = −0.496, *P* < 0.001), *CCL20* (rho = −0.496, *P* < 0.001), and *CCR8* (rho = −0.446, *P* < 0.001). The expression level which was most positively correlated with *CCL14* (rho = 0.273, *P* < 0.001) and most negatively correlated with *CXCL5* (rho = −0.387, *P* < 0.001) was that of *ABCB8*. *ABCC10* showed the most positive correlation with *CXCL14* (rho = 0.345, *P* < 0.001). Considered together, these results revealed that the expression of these five hallmark ABC transporters was closely correlated with immune cell infiltration patterns and immunoregulation in TC.

### Analysis of the Significant Correlations of Hallmark ABC Transporters in TC

**Figure 4:**
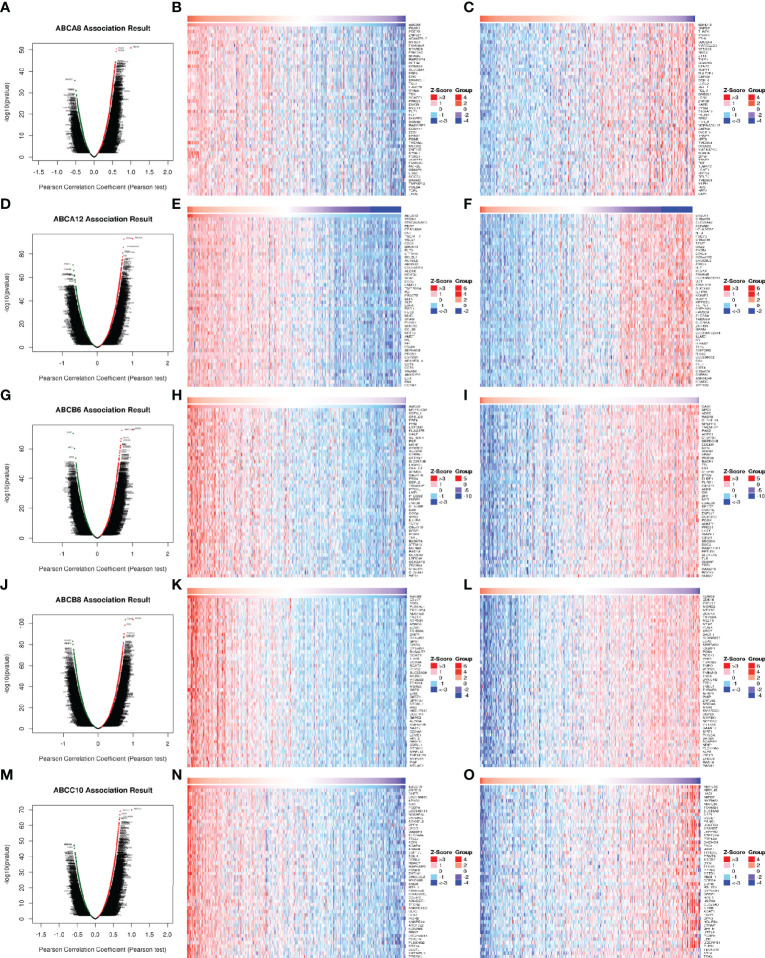
Correlation of differentially expressed genes with five hallmark ABC transporters in TC. **(A, D, G, J, M)** Differentially expressed genes, which were correlated with five hallmark ABC transporters, were analyzed by Pearson test, and visualized in form of volcano plots. **(B, E, H, K, N)** The top 50 genes, which were positively correlated with five hallmark ABC transporters, were visualized in form of heatmaps. **(C, F, I, L, O)** The top 50 genes, which were negatively correlated with five hallmark ABC transporters, were also visualized in form of heatmaps.

To explore the molecular mechanisms underlying the regulation of immune cell infiltration in TC by the five hallmark ABC transporters, we identified significantly correlated genes, using the LinkFinder module of LinkedOmics and visualized them in the form of heatmaps and volcano plots. The results showed that the genes which were most significantly positively correlated with the regulation of immune cell infiltration by *ABCA8*, *ABCA12*, *ABCB6*, *ABCB8*, and *ABCC10* were *platelet endothelial aggregation receptor 1 (PEAR1*; rho = 0.604, *P* < 0.001), *V-set domain-containing T cell activation inhibitor 1* (*VTCN1*; rho = 0.754, *P* < 0.001), *microtubule associated protein 1 light chain 3 alpha* (*MAP1LC3A*; rho = 0.694, *P* < 0.001), *chromosome 2 open reading frame 7* (*C2orf7*; rho = 0.782, *P* < 0.001), and *zinc finger protein 513* (*ZNF513*; rho = 0.682, *P* < 0.001), respectively (**Figure 4**). In addition, the genes that were most significantly negatively correlated with the regulation of immune cell infiltration by *ABCA8*, *ABCA12*, *ABCB6*, *ABCB8*, and *ABCC10* were *abhydrolase domain-containing protein 12* (*ABHD12*; rho = −0.523, *P* < 0.001), *BTB domain containing 11* (*BTBD11*; rho = −0.687, *P* < 0.001), *calpastatin* (*CAST*; rho = −0.687, *P* < 0.001), *lysine demethylase 5B* (*KDM5B*; rho = −0.727, *P* < 0.001), and *mitochondrial ribosomal protein S35* (*MRPS35*; rho = −0.589, *P* < 0.001), respectively (**Figure 4**).

**Figure 5:**
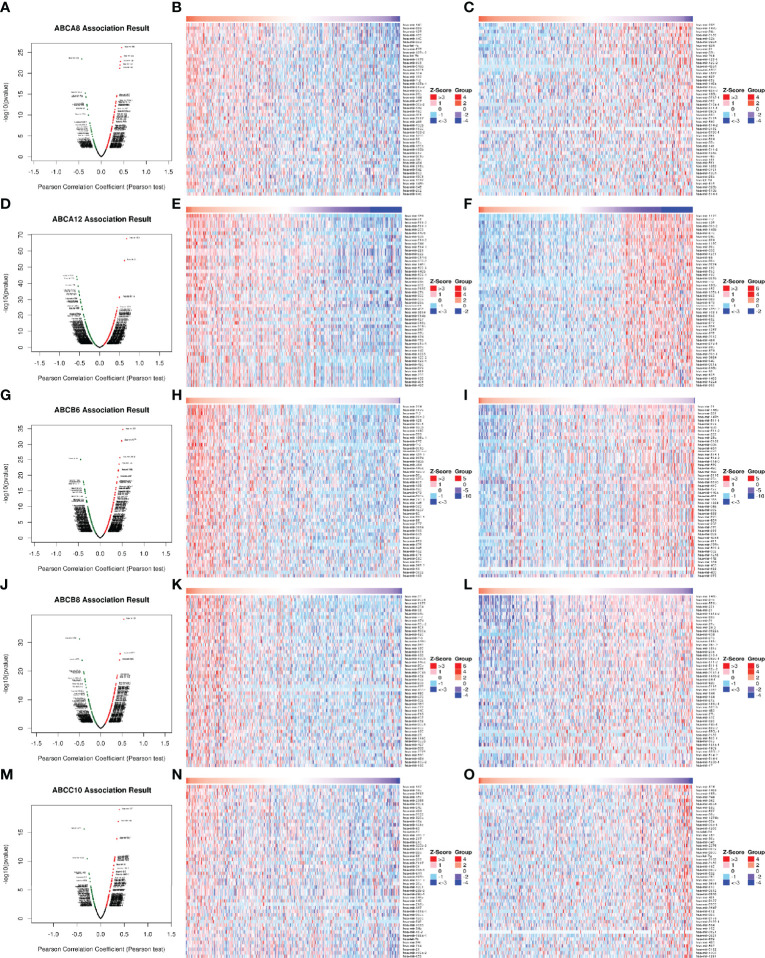
Correlation of differentially expressed miRNAs with five hallmark ABC transporters in TC. **(A, D, G, J, M)** Differentially expressed miRNAs, which were correlated with five hallmark ABC transporters, were analyzed by Pearson test, and visualized in form of volcano plots. **(B, E, H, K, N)** The top 50 miRNAs, which were positively correlated with five hallmark ABC transporters, were visualized in form of heatmaps. **(C, F, I, L, O)** The top 50 miRNAs, which were negatively correlated with five hallmark ABC transporters, were also visualized in form of heatmaps.

It is widely accepted that miRNAs and TFs are key regulators of gene expression. Therefore, we also identified the miRNAs and TF targets that were significantly correlated with the five hallmark ABC transporters, using the LinkFinder and LinkInterpreter modules of LinkedOmics. The results of correlated miRNAs were also present in the form of heatmaps and volcano plots. The miRNAs most significantly positively correlated with *ABCA8*, *ABCA12*, *ABCB6*, *ABCB8*, and *ABCC10* were hsa-mir-145 (rho = 0.454, *P* < 0.001), hsa-mir-934 (rho = 0.677, *P* < 0.001), hsa-mir-204 (rho = 0.517, *P* < 0.001), hsa-mir-22 (rho = 0.538, *P* < 0.001), and hsa-mir-187 (rho = 0.391, *P* < 0.001), respectively (**Figure 5**). Moreover, the miRNAs that were most significantly negatively correlated with *ABCA8*, *ABCA12*, *ABCB6*, *ABCB8*, and *ABCC10* were hsa-mir-203 (rho = −0.432, *P* < 0.001), hsa-mir-1179 (rho = −0.573, *P* < 0.001), hsa-mir-21 (rho = −0.445, *P* < 0.001), hsa-mir-146b (rho = −0.493, *P* < 0.001), and hsa-mir-874 (rho = −0.355, *P* < 0.001), respectively (**Figure 5**). In addition, the TF targets that were most correlated with *ABCA8*, *ABCA12*, *ABCB6*, *ABCB8*, and *ABCC10* were V$LXR_DR4_Q3, V$NFKB_C, V$ELK1_02, V$ELK1_02, and V$PAX8_01, respectively (**Table 4**).

**Table 4:** Correlations of the expression of the five hallmark ABC transporters with the abundance of gene markers of immune cell infiltration in TC.

Cell type	Gene markers	*ABCA12*		*ABCA8*		*ABCB6*		*ABCB8*		*ABCC10*	
		r	*P*	r	*P*	r	*P*	r	*P*	r	*P*
B cell		-0.216	*	0.157	*	0.073	ns	0.08	ns	-0.142	*
	CD19	0.176	*	0.055	ns	-0.148	*	-0.184	*	0.031	*
	CD79A	0.224	*	-0.047	ns	-0.256	*	-0.208	*	-0.012	ns
Th1		-0.147	*	-0.358	*	-0.097	ns	-0.079	ns	-0.288	*
	IFNG	0.159	*	0.027	ns	-0.187	*	-0.175	*	0.063	ns
	STAT1	0.481	*	-0.032	ns	-0.288	*	-0.174	*	0.169	*
	STAT4	0.383	*	-0.035	ns	-0.219	*	-0.270	*	0.189	*
	TNF	0.179	*	0.087	ns	-0.112	*	-0.133	*	0.162	*
	TBX21	0.052	ns	0.105	*	-0.012	ns	-0.064	ns	0.171	*
Th2		0.223	*	-0.198	*	-0.249	*	-0.102	*	-0.12	*
	GATA3	0.316	*	-0.011	ns	-0.073	ns	-0.104	*	0.157	*
	IL13	0.106	*	0.003	ns	-0.085	ns	-0.071	ns	0.124	*
	STAT5A	0.319	*	0.108	*	-0.165	*	-0.088	ns	0.192	*
	STAT6	0.242	*	0.258	*	-0.019	ns	0.016	ns	0.425	*
Tfh		-0.107	*	0.163	*	0.119	*	0.072	ns	-0.06	ns
	BCL6	0.276	*	0.148	*	0.023	ns	-0.039	ns	0.234	*
	IL21	0.070	ns	-0.006	ns	-0.077	ns	-0.079	ns	0.054	ns
Tcm		-0.315	*	0.272	*	0.355	*	0.129	*	0.211	*
Treg		0.403	*	-0.305	*	-0.358	*	-0.275	*	0.106	*
	CCR8	0.417	*	-0.022	ns	-0.274	*	-0.121	*	0.152	*
	FOXP3	0.443	*	-0.101	*	-0.302	*	-0.257	*	0.134	*
	TGFB1	0.129	*	0.342	*	0.184	*	0.105	*	0.519	*
CD8^+^ T		-0.299	*	0.291	*	0.493	*	0.224	*	-0.078	ns
	CD8A	0.074	ns	0.117	*	-0.048	ns	-0.077	ns	0.115	*
	CD8B	0.288	*	0.018	ns	-0.086	ns	-0.224	*	0.212	*
NK		-0.128	*	0.279	*	0.246	*	0.064	ns	-0.129	*
	KIR2DL1	-0.102	*	0.090	ns	0.051	ns	-0.016	ns	-0.013	ns
	KIR2DL3	-0.020	ns	0.069	ns	0.012	ns	-0.042	ns	0.081	ns
	KIR2DS4	-0.100	ns	0.112	*	-0.005	ns	-0.035	ns	0.062	ns
	KIR3DL1	-0.141	*	0.152	*	0.073	ns	0.048	ns	0.102	*
	KIR3DL2	-0.002	ns	0.109	*	0.006	ns	-0.059	ns	0.163	*
	KIR3DL3	-0.067	ns	0.021	ns	-0.062	ns	-0.073	ns	0.017	ns
M1		0.098	ns	0.141	*	-0.137	*	-0.068	ns	-0.111	ns
	IRF5	0.480	*	-0.057	ns	-0.301	*	-0.197	*	0.255	*
	NOS2	0.020	ns	0.191	*	0.106	*	0.179	*	0.219	*
	PTGS2	0.561	*	0.009	ns	-0.277	*	-0.235	*	0.188	*
M2		0.401	*	-0.069	ns	-0.154	*	-0.138	*	0.284	*
	CD163	0.344	*	0.168	*	-0.183	*	-0.061	ns	0.160	*
	MS4A4A	0.352	*	0.090	ns	-0.219	*	-0.163	*	0.126	*
	VSIG4	0.336	*	0.072	ns	-0.276	*	-0.149	*	0.079	ns
Monocyte		0.33	*	-0.166	*	-0.364	*	-0.286	*	0.281	*
	CD86	0.374	*	-0.001	ns	-0.306	*	-0.252	*	0.062	ns
	CSF1R	0.286	*	0.096	ns	-0.173	*	-0.145	*	0.149	*
DC		0.654	*	-0.166	*	-0.432	*	-0.364	*	0.129	*
	CD1C	0.463	*	-0.058	ns	-0.328	*	-0.280	*	0.030	ns
	HLA-DPA1	0.386	*	-0.178	*	-0.406	*	-0.309	*	-0.073	ns
	HLA-DPB1	0.350	*	-0.134	*	-0.344	*	-0.276	*	-0.060	ns
	HLA-DQB1	0.342	*	-0.212	*	-0.377	*	-0.322	*	-0.120	*
	HLA-DRA	0.408	*	-0.166	*	-0.404	*	-0.280	*	-0.061	ns
	ITGAX	0.392	*	-0.030	ns	-0.271	*	-0.252	*	0.206	*
	NRP1	-0.049	ns	0.407	*	0.331	*	0.284	*	0.453	*

Correlations were analyzed using Spearman’s test and adjusted for tumor purity. Th, helper T cell; Tfh, follicular helper T cell; Treg, regulatory T cell; NK, natural killer cell; DC, dendritic cell; r, the purity-adjusted partial Spearman’s rho value. *P < 0.05, ns, not significant.

### Regulation Network Construction and Functional Enrichment Analysis of Hallmark ABC Transporters and Their Correlated Genes in TC

**Figure 6:**
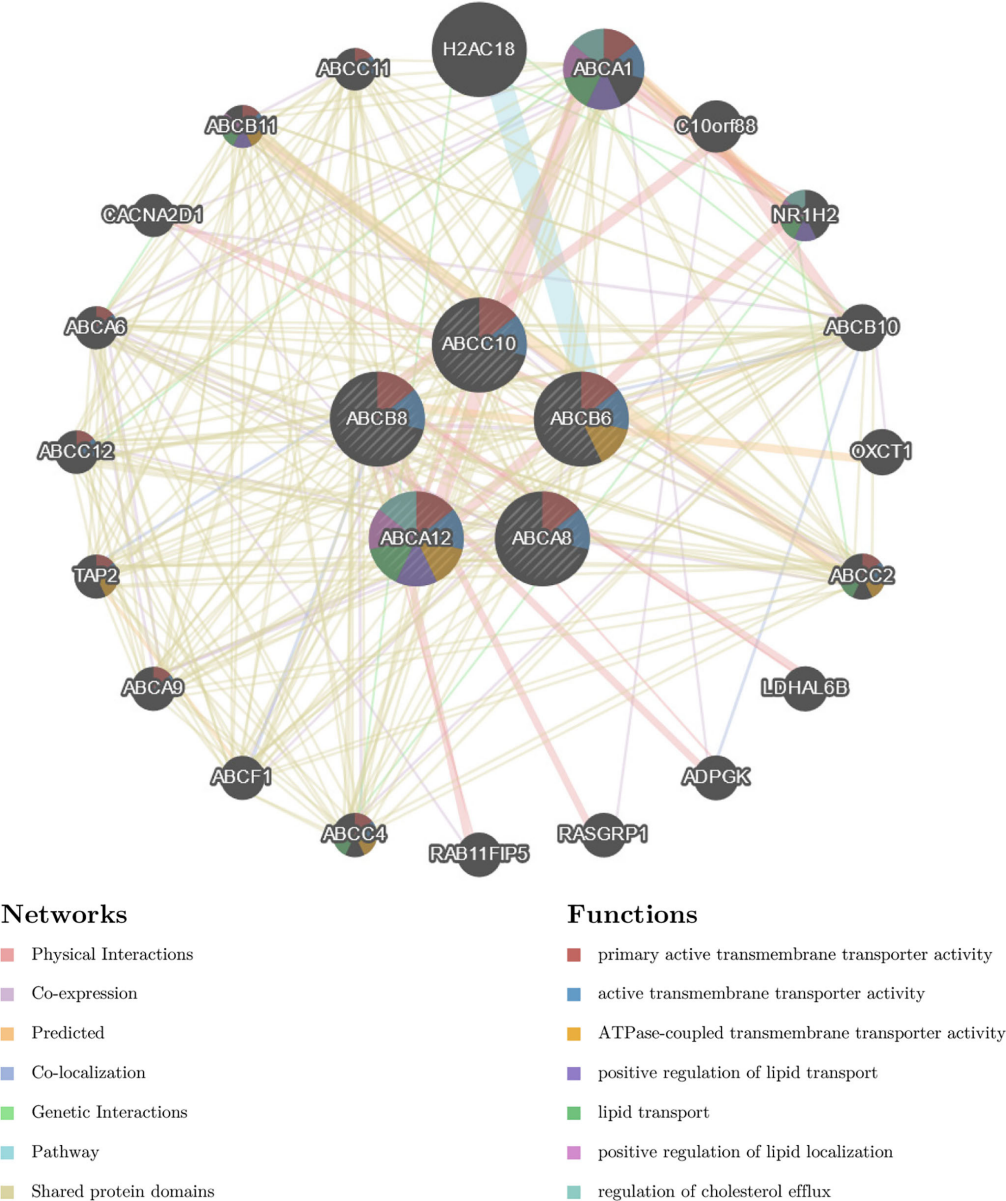
The constructed gene regulation network of five hallmark ABC transporters and 100 genes, which were most highly correlated with five hallmark ABC transporters.

**Figure 7:**
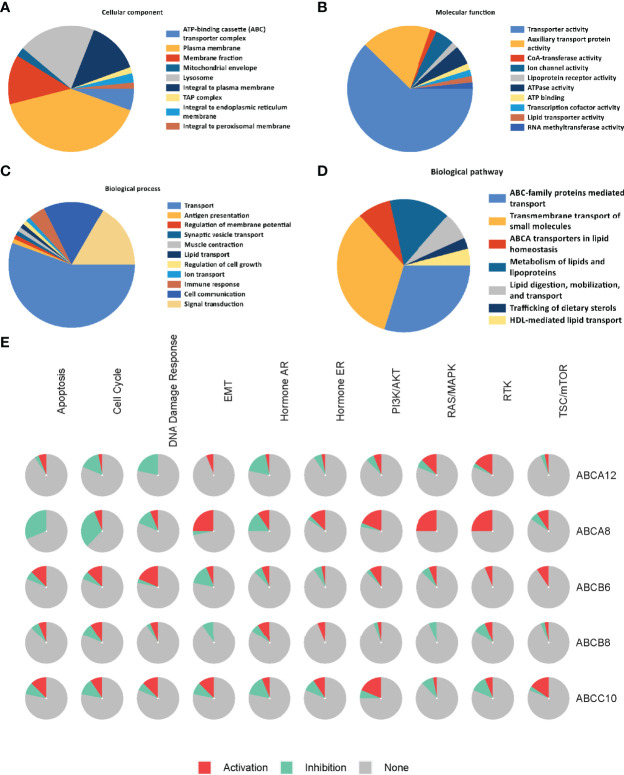
Functional and pathway enrichment analysis of the five hallmark ABC transporters in TC. **(A)** The most enriched cellular components of correlated genes of the five hallmark ABC transporters. **(B)** The most enriched molecular functions of correlated genes of the five hallmark ABC transporters. **(C)** The most enriched biological processes of correlated genes of the five hallmark ABC transporters. **(D)** The most enriched biological pathways of correlated genes of the five hallmark ABC transporters. **(E)** The cancer related pathway enrichment of the five hallmark ABC transporters.

To identify the molecular mechanisms underlying the regulation of immune cell infiltration by hallmark ABC transporters more precisely, we constructed a gene regulation network and conducted functional enrichment analysis of the five hallmark ABC transporters and a 100 of the genes that were most correlated with them, using GeneMANIA and Funrich. The network revealed that these genes were closely associated with transmembrane transporter activity and regulation of lipid transport (**Figure 6**). The enrichment analyses indicated that the most highly enriched cellular components were the plasma membrane (53.7%) and lysosomes (26.3%); (**Figure 7A**). Transporter activity (37.6%) and auxiliary transport protein activity (10.9%) were the most highly enriched molecular functions (**Figure 7B**). The biological processes of genes were most highly enriched in transport (49.5%); (**Figure 7C**). Some biological pathways, including transmembrane transport of small molecules (46.3%) and ABC family protein-mediated transport (40.7%), were also most highly enriched (**Figure 7D**). In addition, cancer process and cancer-related pathway enrichment analyses of these hallmark ABC transporters were performed and visualized using GSCALite. The results showed that all five hallmark ABC transporters were involved in regulating these cancer-related pathways (**Figure 7E**).

### Chemical–Gene Correlation Analysis of Hallmark ABC Transporters in TC

Since hallmark ABC transporters were closely associated with the transmembrane transport of small molecules, we screened out TC-associated chemicals and inferred their association with these five hallmark ABC transporters, using the CTD database. The results showed that multiple types of chemicals, including bisphenol A and vincristine, affected the expression or mutagenesis of these hallmark ABC transporters (**Table 5**). These findings provided important information regarding the nature of interaction between chemicals and hallmark ABC transporters and their effects on the progression of TC.

**Table 5:** Inferred correlation between hallmark ABC transporters and chemicals in TC.

Gene Name	Chemical name	Interaction	References (PubMed ID)
*ABCA8*	Bisphenol A	Bisphenol A results in decreased *ABCA8* expression	29050248; 25181051
*ABCA8*	Doxorubicin	Doxorubicin results in decreased *ABCA8* expression	32173973; 17909728; 16010429; 29803840
*ABCA8*	Indomethacin	Indomethacin results in increased *ABCA8* expression	18791128; 24737281
*ABCA8*	Perfluorooctane sulfonic acid	Perfluorooctane sulfonic acid results in decreased *ABCA8* expression	24420840; 27153767
*ABCA8*	Rosiglitazone	Rosiglitazone results in decreased *ABCA8* expression	17188145; 14736730; 11352223; 25572481
*ABCA8*	Vincristine	Vincristine results in decreased susceptibility to vincristine, which affects *ABCA8* expression	9571977; 19944135
*ABCA12*	1,2-Dimethylhydrazine	1,2-Dimethylhydrazine results in decreased *ABCA12* expression	21864636; 22206623
*ABCA12*	Bisphenol A	Bisphenol A results in both increased *ABCA12* gene methylation and decreased *ABCA12* expression	29050248; 22576693
*ABCA12*	Tretinoin	Tretinoin results in decreased *ABCA12* expression	17045167; 16026305; 23724009
*ABCA12*	Vincristine	Vincristine results in increased *ABCA12* expression	9571977; 23649840; 19944135
*ABCB6*	1,2-Dimethylhydrazine	1,2-Dimethylhydrazine results in decreased *ABCB6* expression	21864636; 22206623
*ABCB6*	4,4’-diaminodiphenylmethane	4,4’-diaminodiphenylmethane results in increased *ABCB6* expression	7505956; 3712494; 6582329; 6587162; 18648102
*ABCB6*	Acrylamide	Acrylamide results in decreased *ABCB6* expression	28606764; 32763439
*ABCB6*	Bisphenol A	Bisphenol A affects *ABCB6* expression	29050248; 29275510
*ABCB6*	Chloroprene	Chloroprene results in increased *ABCB6* expression	23125180; 12562636
*ABCB6*	Copper	Copper results in increased *ABCB6* expression	19497425; 30556269
*ABCB6*	Paclitaxel	Paclitaxel results in decreased *ABCB6* expression	20025538; 20737486
*ABCB6*	Phenobarbital	*ABCB6* gene mutant form results in increased susceptibility to phenobarbital; Phenobarbital results in increased *ABCB6* expression	28245158; 3356011; 3133336; 3137195; 3865012; 3856057; 6850638; 2910521; 19159669
*ABCB6*	Troglitazone	Troglitazone results in increased *ABCB6* expression	15785241; 25596134
*ABCB6*	Vincristine	Vincristine results in decreased susceptibility to vincristine, which affects *ABCB6* expression	9571977; 19944135
*ABCB8*	1,2-Dimethylhydrazine	1,2-Dimethylhydrazine results in increased *ABCB8* expression	21864636; 22206623
*ABCB8*	Bisphenol A	Bisphenol A results in decreased *ABCB8* expression	29050248; 30816183; 25181051
*ABCC10*	2-Acetylaminofluorene	2-Acetylaminofluorene results in increased *ABCC10* expression	28245158; 12566991
*ABCC10*	4,4’-diaminodiphenylmethane	4,4’-diaminodiphenylmethane results in increased *ABCC10* expression	7505956; 1399818; 3712494; 6582329; 6587162; 18648102
*ABCC10*	Acrylamide	Acrylamide results in decreased *ABCC10* expression	28606764; 32763439
*ABCC10*	Bisphenol A	Bisphenol A results in both increased *ABCC10* intron methylation and decreased *ABCC10* expression	29050248; 25181051; 30906313
*ABCC10*	Doxorubicin	Doxorubicin results in decreased *ABCC10* expression	32173973; 17909728; 16010429; 29803840
*ABCC10*	Imatinib mesylate	Imatinib mesylate inhibits the reaction [ABCC10 protein results in decreased susceptibility to paclitaxel and vincristine]	16940797; 19841739
*ABCC10*	Paclitaxel	Paclitaxel results in increased *ABCC10* expression	20025538; 20737486
*ABCC10*	Vincristine	ABCC10 protein results in decreased susceptibility to vincristine	9571977; 19841739

### Prognostic Value of Hallmark ABC Transporters in TC

**Figure 8:**
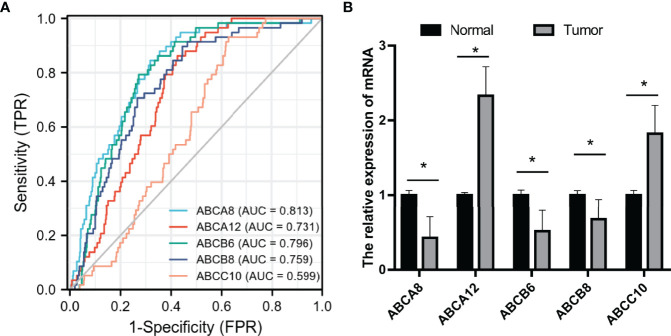
Prognostic prediction value and validation of the five hallmark ABC transporters in TC. **(A)** The ROC curves of the five hallmark ABC transporters in TC. **(B)** Relative mRNA expression of the five hallmark ABC transporters in TC and normal thyroid tissues. FPR, false positive rate; TPR, true positive rate. *P < 0.05.

To evaluate the prognostic value of these five hallmark ABC transporters, ROC curves were generated using the expression data of TC and normal thyroid tissues obtained from TCGA (**Figure 8A**). The AUCs and 95% CI values of these five hallmark ABC transporters were calculated (**Table 6**). Among the five hallmark ABC transporters, *ABCA8* showed the highest relative accuracy while that of *ABCC10* appeared to be the lowest. These results indicated the considerable potential shown by these five hallmark ABC transporters for predicting the RFS of TC.

**Table 6:** Prognostic value prediction of five hallmark ABC transporters in TC.

Variables	AUC	CI	Sensitivity	Specificity
*ABCA8*	0.813	0.763–0.863	84.5%	68.2%
*ABCA12*	0.731	0.681–0.781	86.2%	57.5%
*ABCB6*	0.796	0.747–0.845	79.3%	72.7%
*ABCB8*	0.759	0.703–0.816	89.7%	55.5%
*ABCC10*	0.599	0.540–0.657	93.1%	37.3%

AUC, area under the curve; CI, confidence interval.

### Validation of Hallmark ABC Transporters in TC

Representative hematoxylin and eosin staining images of both collected TC and para-cancerous thyroid tissues were performed (**Supplemental Figure 1**). To validate the bioinformatic analyses of these five hallmark ABC transporters in TC, we performed qRT-PCR tests to examine their transcriptional expression in both TC and para-cancerous thyroid tissues. The results indicated that the expression levels of *ABCA12* and *ABCC10* in TC were higher compared to those in para-cancerous thyroid tissue, while the expression levels of *ABCA8*, *ABCB6*, and *ABCB8* in TC were significantly lower (*P <*0.05; **Figure 8B**). These results were consistent with those obtained from the public *via* TCGA database.

## Discussion

It has become increasingly evident that ABC transporters play important roles in the immunomodulation of tumors. For example, multidrug resistance protein 1 (MDR1), encoded by *ABCB1*, is expressed in cytotoxic T lymphocytes and NK cells, and mediates immune responses (56). A high level of the MDR1^+^ immune cell infiltrate, mostly comprising M2 macrophages, was confirmed as an independent prognostic factor associated with poor prognoses for epithelial ovarian cancer (57). *ABCA1* regulates the immune sensitivity of osteosarcoma cells. Moreover, the *ABCB1*: *ABCA1* ratio was reportedly upregulated in osteosarcoma cells and positively correlated with a higher probability of relapse (58). *ABCA8* expression was downregulated in stomach adenocarcinoma and was positively associated with six types of infiltrated immune cells, particularly M2 macrophages (59). Major histocompatibility complex class I (MHC-I) molecules play a vital role in immune surveillance as well as in the presentation of antigen peptides on the cell surface (60). The heterodimer of transporter associated with antigen processing (TAP) transports antigenic peptides and provides peptides to MHC-I molecules (61, 62). TAP blockade in DCs reportedly impaired classic MHC-I presentation for CD8^+^ T cell priming (63). Moreover, low TAP1/TAP2 expression led to the overexpression and efficient presentation of the antigen preprocalcitonin in lung carcinoma, as recognized *via* tumor-specific cytotoxic T lymphocytes (64). *ABCC8* is considered a prognostic risk factor which is closely related to the LUAD microenvironment (65). Single-nucleotide polymorphisms (SNPs) in *TAP1* and *TAP2* affected their expression and were associated with cervical cancer in the Chinese Han population (66). *ABCD3* expression, which is considered as an independent prognostic factor of CRC, was decreased in CRC tissues and associated with the overall survival of CRC patients (67).

Previous studies have demonstrated that altered expression levels of some ABC transporters were associated with their molecular roles in TC. *ABCA9* expression was upregulated by hsa_circ_0039411 sponging miR-1179, leading to enhanced oncogenic properties in PTC (68). *ABCB5* expression was reported to be associated with larger tumor size in PTC (69). Moreover, in the solid variant of PTC, high *ABCC1* expression was associated with larger tumor size, while high *ABCG2* expression was correlated with lymphovascular invasion (70). *ABCG2* expression was also found to be closely related to the induction of EMT in PTC (71). High *ABCC2* expression was observed in advanced stage MTC (72). In ATC, high expression levels of *ABCB1*, *ABCC1* and *ABCG2* observed in several cell lines and tissues, were associated with cancer drug resistance (73). However, the prognostic and immunotherapeutic value of ABC transporters in TC has not been well characterized. Therefore, the current study aimed to explore the prognostic and immune related value of ABC transporters in TC, leading to the provision of new directions and targets for its treatment.

In this study, data of TC and normal thyroid tissues from TCGA were analyzed to screen out hallmark ABC transporters, which are differentially expressed and associated with the prognosis for TC. Based on the results, *ABCA8*, *ABCA12*, *ABCB6*, *ABCB8*, and *ABCC10* were selected as hallmark ABC transporters in TC. Of these, the expression of *ABCA8*, *ABCB6*, and *ABCB8* was down-regulated in TC, compared with those in normal thyroid tissues, while that of *ABCA12* and *ABCC10* was up-regulated. Usually, up-regulation of oncogenes and down-regulation of anti-oncogenes affect many behavior patterns of malignant tumor cells, including metastasis and immune resistance. Our results revealed that *ABCA8*, *ABCB6*, and *ABCB8* may inhibit the malignant progression of TC. Conversely, *ABCA12* and *ABCC10* may promote the occurrence and growth of TC. Since these are differentially expressed in TC and closely associated with the prognoses for TC, we selected these five as hallmark ABC transporters in TC for subsequent analyses.

It is increasingly becoming evident that immunotherapies play a vital role in tumor treatment, wherein the efficacy of immunomodulation depends mainly on immune cell infiltration. Based on our results, the five hallmark ABC transporters exert a variety of effects on immune cell infiltration in TC. Firstly, the expression of five hallmark ABC transporters was associated with two different TC subsets (hot or cold), indicating that the five hallmark ABC transporters may be useful for converting immunosuppressive (cold) TCs into immunosensitive (hot) ones. Moreover, our results showed that all five hallmark ABC transporters were associated with Th2 cells, Tcm cells, Treg cells, monocytes, and DCs, which revealed that their roles in immune cell infiltration-related immunoregulation were similar. In addition, the specificity of the transporters in regulating Th1 cells, Tfh cells, CD8^+^ T cells, NK cells, M1 macrophages, and B cells demonstrated the diversity of their roles. Our results also indicated that multiple immunomodulators, such as KDR, MHC molecules, VTCN1, CD274, TGFBR1, TNFSF18, and TNFSF15, were closely associated with these five hallmark ABC transporters. KDR, also known as vascular endothelial growth factor receptor 2 (VEGFR2), is the main receptor of VEGF signaling (74). Activation of KDR, which promotes endothelial cell mitogenesis and vascular permeability, plays a vital role in the induction of tumor angiogenesis (75). The VEGF-KDR signaling pathway was found to play an immunosuppressive role in TME and immune effector cell activity (76). MHC molecule expression is known to mediate immune escape mechanisms in tumors (77). VTCN1, also termed B7-H4, reportedly inhibits T cell proliferation and cytokine secretion, thereby negatively regulating T cell immune response, and positively regulating immune escape (78). CD274, also commonly referred to as programmed cell death 1 ligand 1 (PD-L1), is a ligand of PD-1, which is expressed on many immune cells. The PD-1/PD-L1 axis, which is exploited by tumor cells, may inhibit immune response and block immune cell activation (78). Cancer cells generate multiple factors, including TGF-β1, to create an immune inhibitory environment and evade T cell surveillance (79, 80). As the irreplaceable receptor of TGF-β1, TGFBR1, which is observed in different tumor types, participates in tumor immunological reactions (81). Tumor necrosis factor (TNF) superfamily ligands, such as TNFSF15 and TNFSF18, exert diverse modulatory effects by influencing immune responses and impacting immune cells (82–84). Moreover, chemokine–chemokine receptor interactions regulate immune cell recruitment into tumors and the stimulation of immune response (85). Our results demonstrated that the immune regulation by hallmark ABC transporters was partly mediated *via* chemokine ligands and receptors.

The presence of complex regulatory networks that affect almost all molecular processes at both intracellular as well as TME levels, is well known. The results of the analysis conducted on correlated genes, miRNAs, and TF targets showed that each hallmark ABC transporters possessed a unique feature and a distinctive regulatory pattern. The constructed regulation network and enrichment of these five transporters showed that the genes, which were most correlated with the five hallmark ABC transporters, were closely associated with transmembrane transporter activity and ABC family protein-mediated transport. In addition, multiple cancer processes and cancer-related pathways, including RTK, RAS/MAPK, PI3K/AKT, and EMT, were partly activated or suppressed by these five hallmark ABC transporters. These findings indicated that these five hallmark ABC transporters may modulate immune cell infiltration patterns by altering the expression levels of correlated genes, regulating transmembrane transporter activity, and influencing cancer-related pathways. However, the exact regulatory mechanisms have not yet been fully elucidated and further discovery and validation are felt to be required.

The known risk factors for TC include female sex, obesity, smoking status, radioactive iodine exposure history, and family genetic history (86–88). However, these recognized causative factors do not fully explain the increasing incidence of TC. Recent studies have demonstrated the influence of anthropogenic environmental chemical factors on TC (89). For example, individuals with higher cadmium exposure were observed to be more susceptible to TC (90). Environmental radiation exposure, such as that due to the Chernobyl accident, led to radiation dose-associated DNA double-strand breaks, subsequently resulting in PTC growth (91). Artificial light at night has also been found to be positively associated with TC incidence (92). Exposure to multiple essential microelements, including manganese and strontium, were positively associated with capsular invasion, multifocality, and tumor stage of PTC (93). Bisphenol A altered endocrine function and partly facilitated EMT in PTC (94). In this study, we focused on elucidating the associations between TC-associated chemicals and the five hallmark ABC transporters. We indicated that these chemicals exerted significant effects on the expression of these five hallmark ABC transporters in TC. The methylation and mRNA expression levels of these five hallmark ABC transporters were mostly affected by bisphenol A and vincristine. The findings of the present study are consistent with those of previous studies. Therefore, more attention should be paid to regulating the impact of chemical factors on TC.

Moreover, to verify the results of bioinformatic analyses, we performed qRT-PCR to detect the mRNA expression of these five hallmark ABC transporters in collected TC and para-cancerous thyroid tissues. The qRT-PCR results were entirely consistent with our TCGA analysis. In the future, we expect to directly confirm probable mechanisms underlying the regulation of immune cell infiltration patterns in TC by these five hallmark ABC transporters, *via in vitro* and *in vivo* experiments.

Our study was affected by several limitations. Firstly, in our effort to elucidate the specific roles of hallmark ABC transporters in modulating TC prognosis and progression, we analyzed the data of all TCs obtained from the TCGA database, without differentiating between its pathological subtypes. Secondly, compared with PTC, the incidence rates of MTC and ATC are very low. As such, it is relatively difficult to collect ATC and MTC case data for qRT-PCR analysis. Therefore, only PTC and para-cancerous thyroid tissues could be collected and used for experimental validation.

## Conclusions

The findings of this study indicate that five hallmark ABC transporters (*ABCA8*, *ABCA12*, *ABCB6*, *ABCB8*, and *ABCC10*) are strongly associated with immunomodulation in TC, as well as with the prognoses for TC. In addition, factors, as well as chemicals and regulatory networks, that are significantly correlated with these five hallmark ABC transporters in TC, are elucidated. These findings may help better understand the molecular mechanisms underlying the role played by these hallmark ABC transporters in TC. The findings of this study also indicate that these hallmark ABC transporters may help enhance prognostic prediction and enable the development of effective immunotherapies against TC.

## Data Availability Statement

The original contributions presented in the study are included in the article/**Supplementary Material**. Further inquiries can be directed to the corresponding author.

## Ethics Statement

The studies involving human participants were reviewed and approved by the Ethics Committee of Shengjing Hospital of China Medical University. The patients/participants provided their written informed consent to participate in this study.

## Author Contributions

Conceptualization, LW and ZL; Methodology, LW, XS and JH; Investigation, LW and XS; Resources, LW, XS and JH; Writing – Original Draft Preparation, LW; Writing – Review & Editing, XS, JH and ZL; Supervision, ZL. All authors read and approved the final manuscript.

## Funding

This work was supported by the National Natural Science Foundation of China (Grant number: 81672644), the Young Scholar Support Program 2018 of China Medical University (Grant number: QGZD2018061), and the 345 Talent Project of Shengjing Hospital of China Medical University (50A; 30).

## Conflict of Interest

The authors declare that the research was conducted in the absence of any commercial or financial relationships that could be construed as a potential conflict of interest.

## Publisher’s Note

All claims expressed in this article are solely those of the authors and do not necessarily represent those of their affiliated organizations, or those of the publisher, the editors and the reviewers. Any product that may be evaluated in this article, or claim that may be made by its manufacturer, is not guaranteed or endorsed by the publisher.

## Supplementary Material

Supplementary Figure 1 Representative hematoxylin and eosin staining images of both collected TC and para-cancerous thyroid tissues. **(A)** Representative hematoxylin and eosin staining image of para-cancerous thyroid tissues. **(B)** Representative hematoxylin and eosin staining image of PTC tissues. Magnification 200×.
