# Supplementary figures and images for: Identification and Validation of Prognostic Related Hallmark ATP-Binding Cassette Transporters Associated With Immune Cell Infiltration Patterns in Thyroid Carcinoma
Source: Front Oncol. 2022 Jun 28;12:781686. doi: 10.3389/fonc.2022.781686 (PMC9273952; doi:10.3389/fonc.2022.781686)

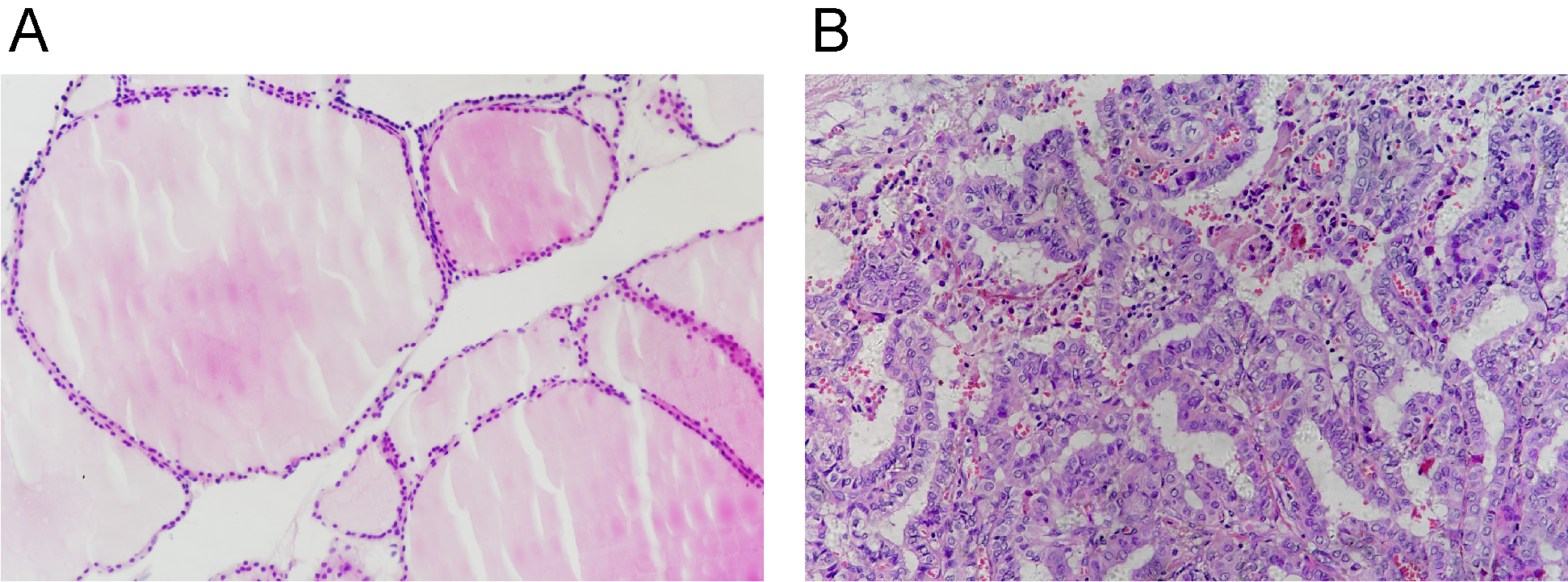

Supplement: Supplementary Figure 1 — Representative hematoxylin and eosin staining images of both collected TC and para-cancerous thyroid tissues. (A) Representative hematoxylin and eosin staining image of para-cancerous thyroid tissues. (B) Representative hematoxylin and eosin staining image of PTC tissues. Magnification 200×. [file Image_1.tif]
